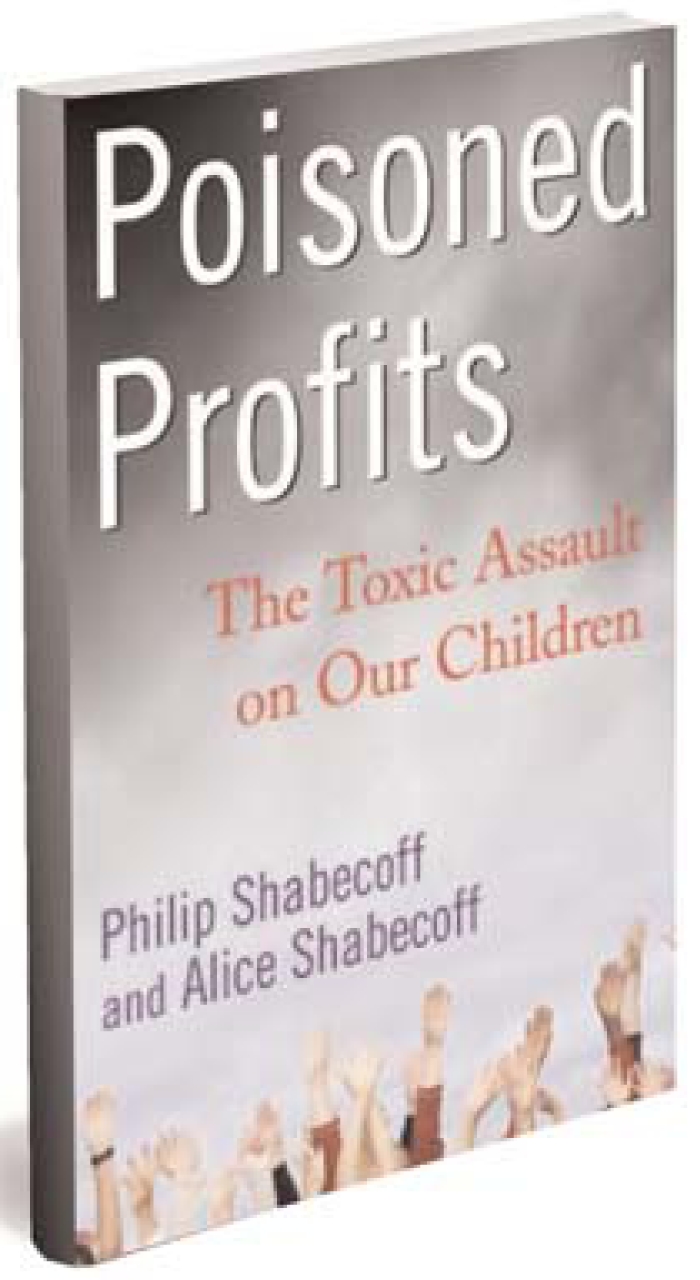# Poisoned Profits: The Toxic Assault on Our Children

**Published:** 2008-10

**Authors:** Ruth A. Etzel

**Affiliations:** Ruth A. Etzel is with the Department of Environmental and Occupational Health at George Washington University School of Public Health and Health Services. She is the editor of Pediatric Environmental Health, published by the American Academy of Pediatrics

An increasingly vast body of evidence shows that some chronic conditions such as birth defects, cancers, and developmental disorders among children are linked to the poisons that are dumped into the food children eat, the water they drink, and the air they breathe. Children are especially vulnerable to the effects of many chemicals, but industrial chemicals are rarely tested for health and safety before sale. Although many birth defects, cancers, and developmental disorders are preventable, neither government nor industry is taking the steps needed to prevent them. In their new book, Philip and Alice Shabecoff argue convincingly that this is a scandal and a crime.

The scope of the problem is huge. One indicator is that filing cabinets at the Centers for Disease Control and Prevention and state health departments contain hundreds of files with details of cluster investigations. Complaints to these agencies called in by ordinary citizens prompted many of these investigations. In some cases, parents believed that more than the usual number of children in their community had a certain birth defect or cancer and were concerned that it might be caused by releases of chemicals from local manufacturers. Frequently no one investigated their complaints at all; when a complaint was investigated, the work was often minimal and the science inconclusive. The reports rarely received public attention.

This solemn and hard-hitting exposé, written for the layperson, explains that the limits of these cluster investigations and other research efforts are no accident. The authors detail the ways in which the petrochemical industry, now the largest manufacturing sector in the United States, influences government science and the process of decision making. The authors contend that because of increasing financial ties between academic researchers and industry, so-called independent scientists and their findings also can be tainted.

The Shabecoffs put a human face to each cluster, introducing us to Peyton, one of the 19 children born with a cleft lip and palate in during a 2-year period in the late 1990s in Dickson, Tennessee, population 13,000. Many of the Dickson parents believed that the birth defects occurred because the mothers drank groundwater contaminated with trichloroethylene (TCE). Some of the townspeople liked the taste of the water (one woman was considering bottling it to sell)—until they learned that the sweet taste (and the cluster of defects) were likely linked to maternal exposures to TCE, dumped there by a company that manufactured automobile parts.

The authors contend that Peyton and the other 18 children in Dickson, and many other children with certain chronic disorders, are victims of an insidious crime. *Poisoned Profits* chronicles the indictment, describes the victims, outlines the evidence, and explores the scene of the crime, including forensics, perpetrators, co-conspirators, witnesses for the defense, and the posse, a group of ordinary people who take justice into their own hands. The parents of affected children, the Shabecoffs, explain, find themselves thrown into an unruly marketplace of information and ideas where they struggle to evaluate evidence they often don’t know how to judge. This the authors distinguish from the world of orthodox science and medicine. The public and private institutions charged with the welfare of children fail to protect them and sometimes do not even listen to the parents’complaints. It is up to courageous doctors, scientists, parents, advocates, and ordinary citizens to embark on a mission to obtain justice for the children. But should the public have to do this on its own? And will enough people do so?

The jury is still out on whether the public will demand effective changes in environmental policy and corporate practices that permit the increasing level of toxicity in our society. After reading this well-documented and accessible analysis, which exposes the American institutions’ willingness to ignore science and public health in favor of protecting corporations’ rising profit rates, many will be roused to action.

## Figures and Tables

**Figure:**